# Neural substrates associated with irrelevant information suppression in problem-solving: an fMRI study of the Remote Associates Test

**DOI:** 10.3389/fnhum.2025.1607193

**Published:** 2025-08-21

**Authors:** Reiji Ohkuma, Yuto Kurihara, Rieko Osu

**Affiliations:** ^1^Graduate School of Human Sciences, Waseda University, Tokorozawa, Japan; ^2^Faculty of Human Sciences, Waseda University, Tokorozawa, Japan

**Keywords:** problem-solving, fMRI, Remote Associates Test, distractor, suppression

## Abstract

Suppressing irrelevant information during problem-solving is vital. Misleading or unrelated information may hinder the performance. However, previous studies inferred suppression-related brain regions based on overall problem-solving or pre-solution neural activity, resulting in insufficient experimental control over the precise timing of suppression and the types of information requiring suppression. In this study, we presented different types of distractors when introducing a problem to examine neural activity associated with suppressing unnecessary information. Participants completed the Japanese version of the Remote Associates Test in an MRI scanner under three conditions: strongly misleading, weakly related, and no distractors. Before the experiment, the participants were informed about the distractors and instructed to ignore them when the problem was presented. The findings showed that stronger suppression demands at problem onset increased the activation of the bilateral inferior frontal gyrus (IFG). Furthermore, the IFG activity, initiated at the beginning of the problem, decreased gradually rather than toward obtaining a solution to the presented problem. These findings suggest that the bilateral IFG supports problem-solving by suppressing irrelevant information.

## 1 Introduction

During problem-solving tasks, such as the Remote Associates Test (RAT), which evaluates creativity, suppressing irrelevant information is critical; failure to do so may impair performance. For example, introducing misleading information reduces performance compared to conditions without such distractors (Jung-Beeman et al., 2004; Metcalfe and Wiebe, 1987).

Recent research suggests that these suppression processes involve the right dorsolateral prefrontal cortex (DLPFC), inferior frontal gyrus, and posterior parietal cortex (Becker et al., 2020b; Tang et al., 2016). Tang et al. (2016) employed a chunk decomposition task using Chinese characters and reported that the bilateral inferior frontal gyri, posterior parietal cortex, and right DLPFC were involved in breaking down existing chunks and suppressing unnecessary information. In addition, Becker et al. (2020b) used a compound remote association task and showed increased activation in the left inferior frontal gyrus (IFG) when non-target semantic associations were suppressed compared with target associations.

These studies, however, have not measured neural activity at the precise moment when unnecessary information is presented. Instead, suppression-related regions have been inferred from brain activity throughout the problem-solving process or immediately before reaching a solution (Becker et al., 2020b; Tang et al., 2016). Therefore, the specific brain activity at the moment when information should be suppressed remains to be determined. In addition, the effects of varying suppression intensities on neural activity have not been fully elucidated. Real-world problem-solving involves strongly and weakly misleading information, potentially engaging in gradually different cognitive mechanisms. However, only a few studies have examined these differences in detail.

This study aimed to clarify the neural substrates underlying the suppression of unnecessary information during problem-solving. To this end, we employed the Japanese version of the Remote Associates Test (RAT) using kanji characters and measured brain activity by functional magnetic resonance imaging (fMRI). Three experimental conditions were established: (1) strongly misleading distractors forming incorrect kanji idioms and evoking incorrect semantic networks; (2) weakly misleading distractors that do not produce meaningful kanji idioms; and (3) no distractors. We manipulated the strength of suppression using these three distractor conditions and examined the resulting differences in the neural activity. By comparing (1) and (2), we determined how neural activity was influenced by the strength of the distractors. By comparing (2) and (3), we identified the effects of the existence of distractors. By comparing (1) and (3), we could infer the combined impact of the strongly misleading distractors. By simultaneously presenting distractors with the problem and instructing participants to ignore them when they appeared, we controlled the timing of the suppression events, enabling a more detailed analysis of the associated neural activity under varying suppression levels.

## 2 Materials and methods

### 2.1 Participants

Twenty-two university students (12 males, 10 females; mean age = 21.9 ± 1.88 years; all native Japanese speakers) from Waseda University participated in the study. The MRI scan of one male participant stopped unexpectedly during the experiment, and the head movement of another male participant exceeded 4 mm during scanning; thus, both were excluded from the analysis, resulting in a final sample of 20 participants. All participants were right-handed, had normal or contact-corrected vision, and had no history of neurological or psychiatric disorders. All participants provided written informed consent before participating in the experiment. The experimental procedures were approved by the Ethics Review Committee of Waseda University (2022-157). All experiments were conducted in accordance with relevant guidelines and regulations.

### 2.2 Materials

We employed the Remote Associates Test (RAT) as a task requiring the suppression of unnecessary information during fMRI (Mednick, 1962; Wu et al., 2020). In the English version of the RAT, participants were presented with three words and asked to generate a common word that could form a compound phrase or idiom for each word. For example, given the words “Pure,” “Blue,” and “Fall,” participants would respond with “Water” (forming “Pure water,” “Blue water,” and “Waterfall,” respectively). This task involves divergent thinking (idea generation), convergent thinking (idea evaluation), and non-insightful analytical thinking (Sowden et al., 2015; Wu et al., 2016, 2017). This study employed the Japanese version of the RAT, which required participants to create two-character kanji idioms (Terai et al., 2013). In this version, participants were given the first character of three different two-kanji idioms and had to generate the common second character to complete each idiom.

**FIGURE 1:**
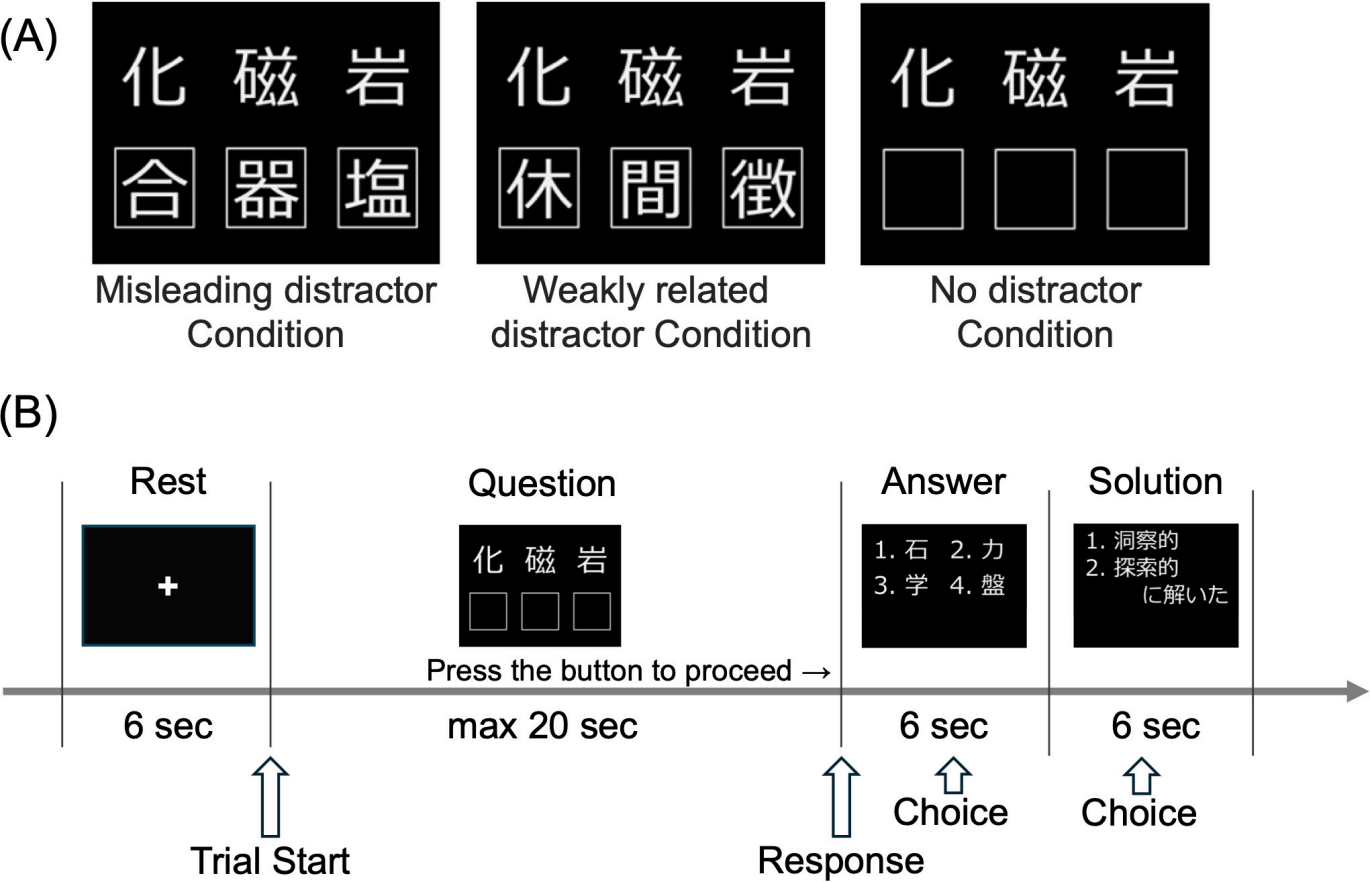
Overview of the Japanese Version of the Remote Associates Test (RAT). **(A)** Three conditions of RAT are presented. Participants are presented with three kanji characters in the upper row and must determine which common kanji character can form a compound idiom with each character. Based on the condition, a distractor may be presented in the lower box of the figure. The correct answer for the example problem is “Ishi” (“ka-seki,” “ji-shaku,” “gan-seki”). Left: In the Misleading Distractor Condition, the distracting kanji character forms an idiom with each of the upper kanji, although it is not the correct answer (e.g., “ka-gou,” “ji-ki,” “gan-en”). Center: In the Weakly Related Distractor Condition, the distracting kanji character does not form an idiom and is weakly related to the given kanji. Right: In the No Distractor (control) Condition, no distracting kanji character is introduced. **(B)** A representation of a single RAT trial. One of the three conditions was presented during the question phase. Participants pressed a button upon finding the answer. In the Answer phase, participants selected the correct answer. Six seconds later, the participants categorized their thought process as “insightful” or “exploratory.” Events for analysis were defined as follows: the beginning of the Question phase as “Trial Start,” the moment the participant pressed the button upon finding the answer as “Response,” and button presses during the Answer and Solution phases as “Choice.”

We investigated neural activity associated with the suppression of unnecessary information at varying intensities by manipulating the presentation of distractors (Figure 1A). We used the 79-item Japanese Remote Associates Test developed by Terai et al. (2013). In the misleading distractor condition, the distractor kanji formed an idiom with the initial kanji but did not yield the correct answer. In the weakly related condition, the distractor kanji were only weakly associated with the initial kanji. In the no-distractor condition, only the initial kanji were presented. Terai et al. (2013) administered the Remote Associates Test to 41 participants under misleading and weakly related conditions and reported the response times and accuracy for each condition. We selected 60 items with shorter response times in the misleading condition because of the time limit of the fMRI experimental paradigm. For each participant, we used a randomization sequence to assign these items to three conditions, with 20 items per condition. To confirm that difficulty was balanced among the three conditions for each participant, we compared the average response times and accuracy of the problems across the three conditions assigned to each participant using the data from Terai et al. (2013). We performed a one-way repeated-measures ANOVA for each participant. The analysis showed no significant differences across conditions for all participants (all *p* > 0.4). This confirms that our random assignment produced equivalent difficulty profiles.

Each trial consisted of four phases: rest (6 s), question (maximum 20 s), answer (6 s), and solution (6 s) (Figure 1B). During the Rest phase, participants viewed a black background with a white fixation cross and were instructed to relax without engaging in cognitive processing. In the Question phase, a RAT question with one of three distractor conditions was randomly presented, and participants were instructed to press a button with their right hand immediately when arriving at an answer. Upon pressing the button, the participants immediately proceeded to the answer phase. In the Answer phase, the participants were presented with four kanji character options and instructed to select the correct option. Each set of options included the correct answer. In the Solution phase, participants were asked to classify their thought processes as “insightful” or “exploratory.” Prior to the task, the participants were informed that “insightful solutions” referred to those reached through sudden realization without conscious reasoning, whereas “exploratory solutions” involved deliberate problem-solving strategies, such as using syllabary order to systematically derive the answer. If participants failed to arrive at an answer within 20 s, they automatically advanced to the Answer and Solution phases. In these cases, they were instructed to disregard the question and refrain from making any selections. Similarly, if participants had an answer in mind but found that their anticipated answer was not among the presented options, they were instructed to disregard the question and not respond. Theoretically, participants could have potentially used an elimination strategy by waiting until the Answer phase and selecting an answer from the presented options. However, the sequential presentation of problems and options, duration of only 6 s for the Answer phase, and the instruction not to answer when they failed to find the answer during the Question phase likely limited the application of the elimination strategy. Each session consisted of 20 trials with an equal distribution of the three conditions. Each participant underwent three sessions. Before the experiment, we instructed the participants that the three distractor conditions would be randomly presented in each session and that they should ignore the distractors and identify the target kanji as quickly as possible.

### 2.3 Imaging acquisition

Functional magnetic resonance imaging was performed using a GE SIGNA Premier with 48 channel coils at 3T. Sequence parameters were used with multiband, which enabled simultaneous imaging of multiple cross-sections to obtain functional images (gradient-echo echo-planar imaging (EPI); repetition time (TR) = 1,000 ms; echo time (TE) = 25 ms; flip angle = 90°; matrix = 64 × 64; 38 axial slices of 3.5 mm thickness with gapless; field of view = 224 × 224 mm; voxel size = 3.5 × 3.5 × 3.5 mm). T1-weighted high-resolution anatomical images were acquired for each participant before obtaining functional images (MP-RAGE; TR = 2171.6 ms; TE = 3.404 ms; TI = 900 ms; FOV = 224 mm × 224 mm; FA = 8°; matrix = 280 × 280; 232 slices with gapless; voxel size = 0.8 × 0.8 × 0.8 mm).

### 2.4 Behavioral analysis

We analyzed trials in which participants pressed the response button within 20 s during the question phase and selected the correct option. The response times for correctly answered problems and the number of correct responses were calculated for each condition. A one-way repeated-measures ANOVA with three conditions was conducted for each measure. When significant differences were observed, we conducted *post hoc* paired-samples *t*-tests between each pair of conditions with Bonferroni correction (adjusted α = 0.05/3) to control for the family wise error rate.

### 2.5 Imaging analysis

All fMRI data analyses were performed using SPM12 (Welfare Department of Cognitive Neurology, London, UK) (Penny et al., 2006). The first five scans of each fMRI experiment, taken for the MR signal to reach equilibrium, were deleted. All functional images were subjected to slice-timing correction, which was spatially corrected for head motion and temporally corrected at the 19th scan, centered at 38 scans/s. The corrected functional and T1-weighted structural images were normalized to a standard T1 template image (ICBM 152) in Montreal Neurological Institute (MNI) space. The parameters from this normalization process were applied to each functional image. The normalized functional images were smoothed using a Gaussian kernel with a full width at half maximum (FWHM) of 8 mm on the *x*, *y*, and *z* axes.

First-level analyses were conducted using a general linear model (GLM) framework in SPM12. At the first level, individual data were analyzed using event-related GLM (Table 1). “Trial Start” events were modeled from the moment the problem was presented (0 s) for a duration of 3 s to capture neural activity related to information suppression. This 3 s window was selected in line with Becker et al. (2020a), who demonstrated that early suppression-related activation is most pronounced during the first 3 s post-stimulus. “Answer Choice” and “Solution Choice” events were modeled at the exact button press to exclude response related activity. Only correctly answered trials were included in the onset regressors for each condition and button press response. As participants were assumed not to have predetermined strategies at trial onset, the analyses were not differentiated by insightful or exploratory solutions. Each event was convolved with a canonical hemodynamic response function (HRF). Six head motion parameters were included as nuisance regressors, and a high-pass filter (128 s cutoff) was applied. Beta estimates were calculated, and first-level contrast images were generated for each participant.

**TABLE 1:** Types of onsets incorporated into general linear model (GLM).

Phase	Event type	Duration (s)	Description
Trial start	1. Misleading distractor condition	3	Information suppression activities
	2. Weakly related distractor condition	3	Information suppression activities
	3. No-distractor condition	3	Information suppression activities
Response -2 s	4. Misleading distractor condition	2	Resolution activities
	5. Weakly related distractor condition	2	Resolution activities
	6. No-distractor condition	2	Resolution activities
Answer choice	7. Button pushing	0	Response related activities
Solution choice	8. Button pushing	0	Response related activities

Listed is the classification of the phases (Trial Start, Response, Answer Choice, and Solution Choice) along with their respective event types.

At the second level, separate random-effects analyses were performed for trial start and response onset. For each onset type, a repeated-measures ANOVA was conducted with three conditions (misleading, weakly related, and no distractor). When a significant cluster was identified, contrast values were extracted at the peak coordinates, and pairwise paired *t*-tests were conducted with FDR correction for multiple comparisons. Specifically, we examined the following contrasts: misleading vs. no distractors, weakly related vs. no distractors, and misleading vs. weakly related distractors. We adopted a cluster-level FWE correction at *p* < 0.001 for all resulting statistics from the whole-brain analyses. Statistical maps were overlaid on the average normalized structural images of all participants. Anatomical regions were determined using the AICHA atlas based on peak voxels from a random effects analysis (Joliot et al., 2015).

To determine how clusters showing significant differences between conditions changed over time, we extracted BOLD signal change rates within a 5 mm radius sphere centered on the statistically significant peak coordinates of each cluster using MarsBar.^1^ The BOLD signal change rates were extracted for each participant in each condition and averaged across all participants, with the baseline set at 0 s before the problem was presented.

## 3 Results

### 3.1 Behavioral results

**FIGURE 2:**
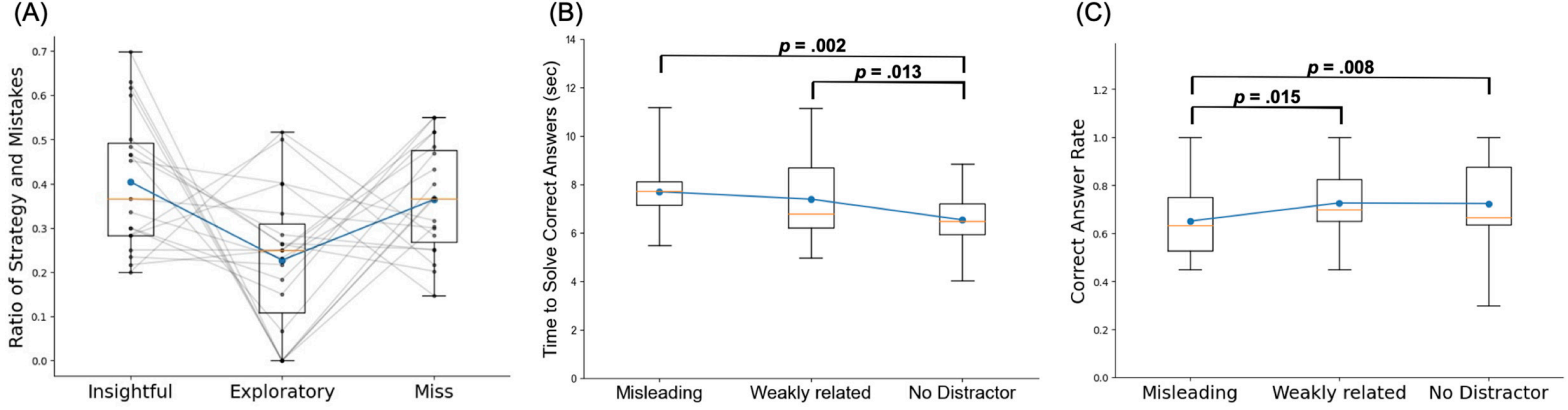
Task performance. **(A)** Ratio of strategy (insightful or exploratory) and mistakes. The black lines represent each subject. **(B)** Response times for correctly answered RAT problems. **(C)** Accuracy rates. A repeated-measures ANOVA was conducted: if significant effects were observed, multiple comparisons were adjusted using Bonferroni correction. The orange lines and blue dots indicate the median and average, respectively.

One participant was excluded from further analysis because the average response time on the RAT task was 1.75 s (group mean ± SD: 6.95 ± 2.61 s), as the short interval between Trial Start and Response made it unsuitable for neural analysis. Consequently, behavioral performance and neural activity were evaluated in a final sample of 19 participants. Regardless of the distractor condition, we assessed strategy use by plotting the number of trials for each strategy (Figure 2A). Individual differences in strategy use resulted in a limited number of trials per condition for some participants, which prevented separate analyses of brain activity by strategy.

Response times for correctly answered problems differed significantly among the three conditions [F(2,36) = 6.39, *p* = 0.005; Figure 2B]. Multiple comparisons showed a significant difference between the misleading distractor and no-distractor conditions [t(18) = 3.48, *p* = 0.002], and a trend-level significance between the weakly related distractor and no-distractor conditions [t(18) = 2.74, *p* = 0.013]. No significant difference was observed between the misleading and weakly related distractor conditions [t(18) = 0.833, *p* = 0.416].

Accuracy rates differed significantly among the three conditions [F(2,36) = 4.13, *p* = 0.024; Figure 2C]. Multiple comparisons revealed a significant difference between the misleading and weakly related distractor conditions [t(18) = 2.96, *p* = 0.008] and a trend-level significance between the misleading distractor and no-distractor conditions [t(18) = 2.67, *p* = 0.015]. No significant difference was observed between the weakly related distractor and non-distractor conditions [t(18) = 0.070, *p* = 0.945].

### 3.2 Imaging results

**FIGURE 3:**
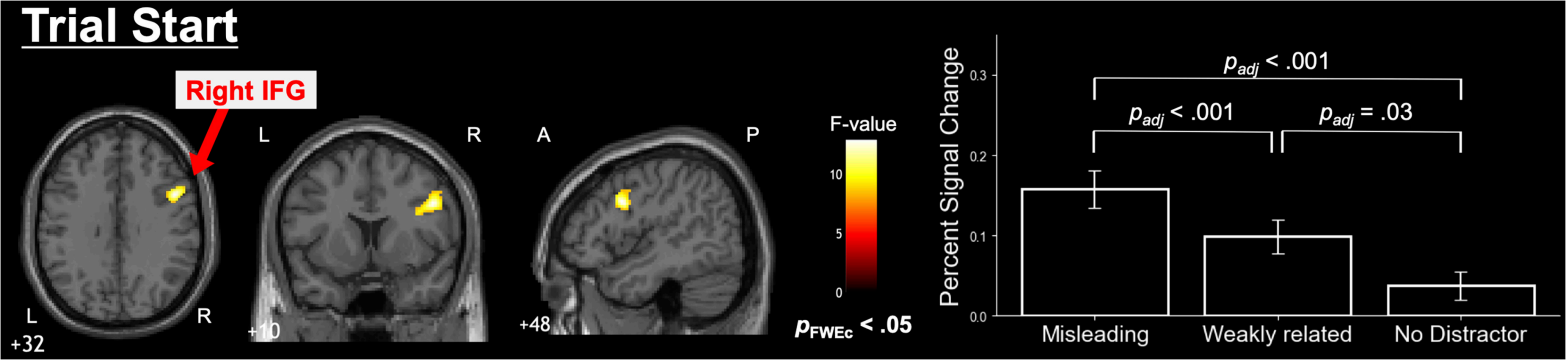
Neuroimaging results from the three-condition comparison. During trial onset, repeated-measures ANOVA detected a significant cluster in the right inferior frontal gyrus (IFG). *Post hoc* multiple comparisons of cluster peak coordinates revealed the following relationship: misleading distractor > weakly related distractor > no distractor. Statistical significance was corrected using false discovery rate. No significant differences were observed in response onset analysis.

At the start of the trial, significant differences in right IFG activity were observed across the three conditions (Figure 3 and Table 2). *Post-hoc* tests on the contrast values at the peak coordinates within the cluster revealed the following relationship: misleading distractor > weakly related distractor > no distractor. No significant condition-related differences were observed in the response onset.

**TABLE 2:** Cluster information for the three-condition comparison (fMRI results during trial start: repeated - measures ANOVA comparing the three conditions).

Anatomical areas	Side	Peak voxel MNI coordinates (mm)			Cluster size (voxel)	Peak *F* score	*P*-value (FWEc)
		*x*	*y*	*z*			
Inferior frontal gyrus	Right	48	10	32	243	12.9	0.050

Shown are the clusters identified in Figure 3 with cluster-level FWE correction applied. The *F*-values represent the peak coordinates of each cluster. Anatomical regions were identified using the AICHA atlas, and MNI refers to the Montreal Neurological Institute (MNI) standard space. No significant condition-related differences were observed in the response onset.

**FIGURE 4:**
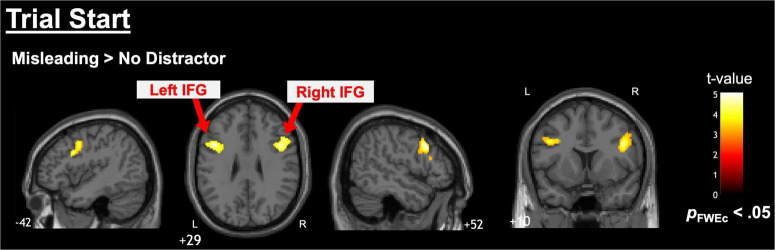
Neuroimaging results from pairwise comparisons. Pairwise comparisons at the start of the trial revealed significant differences between the misleading and no-distractor conditions, showing greater activation in the right and left IFG. No significant differences were found in the other condition comparisons or response onset analyses.

Pairwise comparisons (misleading distractors vs. weakly related distractors, misleading distractors vs. no distractors, and weakly related distractors vs. no distractors) at the start of the trial indicated that misleading distractors elicited greater activation than no distractors in the right and left IFG (Figure 4 and Table 3). No other comparisons showed significant differences, and no significant differences were observed at the response onset.

**TABLE 3:** Cluster information from pairwise comparisons (fMRI results during trial start: Misleading distractor > No distractor).

Anatomical areas	Side	Peak voxel MNI coordinates (mm)			Cluster size (voxel)	Peak *t* score	*P*-value (FWEc)
		*x*	*y*	*z*			
Inferior frontal gyrus	Left	−42	12	30	432	5.10	0.015
Inferior frontal gyrus	Right	44	10	28	582	4.86	0.004

Shown is the cluster information identified in Figure 4 with cluster-level FWE correction. The *F* values represent the peak coordinates of the cluster. Anatomical regions were determined using the AICHA atlas, and MNI coordinates referred to the Montreal Neurological Institute standard space. No other comparisons showed significant differences, and no significant differences were observed at the response onset.

**FIGURE 5:**
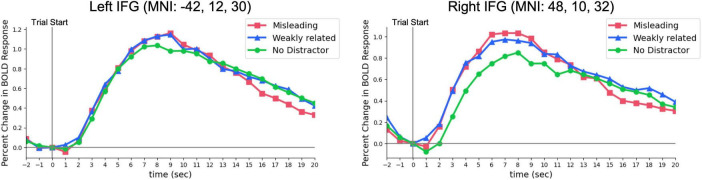
Time course of BOLD signals in the right and left IFG from problem onset (0 s). The BOLD signals in the right and left IFG gradually decreased after peaking. A sphere with a radius of 5 mm was defined around the statistically significant peak coordinates within each cluster, and the mean BOLD signal change was extracted. However, since the problem-solving duration varied across trials, caution must be taken since participants were not engaged in problem-solving for the entire duration.

The BOLD signals in the right and left IFG increased immediately after the onset of the problem, peaking at approximately 6 s (Figure 5) and then gradually declining rather than decreasing abruptly.

## 4 Discussion and conclusion

In this study, we examined brain activity related to irrelevant information suppression using fMRI under three RAT conditions: strongly misleading, weakly related, and no distractors. Previous research suggests that strongly misleading distractors delay the suppression of irrelevant information, thereby hindering the transition to a correct representation and reducing accuracy (Knoblich et al., 1999, 2001). Therefore, in the present study, we manipulated the distractors to modulate the strength of the suppression. As expected, we observed differences in response time and accuracy across the three conditions. Although brain activity differed at the onset of the problem, no differences in brain activity were observed at the solution stage, indicating that the problem and presented distractor influenced the early stages of problem-solving, while their effects diminished in the later stages.

In this study, the finding that the right IFG becomes more active under conditions requiring strong distractor suppression is consistent with previous research indicating that the right IFG inhibits erroneous associations and strong biases, specifically, the stronger the presumed semantic information, the more crucial the right IFG suppression mechanism (Anderson et al., 2009; Shen et al., 2013). As shown in Figure 5, bilateral IFG activation also occurs in the no distractor condition, indicating that the IFG supports the language-dependent insight and working memory processes inherent to the RAT. Furthermore, the stronger right IFG activation for the Misleading > Weakly Related contrast and left IFG for the Misleading > No-Distractor contrast may reflect an expanded semantic search space under strongly misleading distractors, in line with (Becker et al., 2020b). Conversely, the significant increase in the right IFG for Weakly Related > No-Distractor suggests the engagement of selective attention mechanisms to ignore weakly related distractors (Smith and Blankenship, 1991). These findings may indicate that IFG activation in the No-Distractor condition indexes top-down suppression of distractor-related semantic information, which recruits language-based working memory resources during RAT performance.

In the early stages of problem-solving, it is crucial to suppress the activation of knowledge irrelevant to the goal while exploring appropriate information (Lv, 2015). To achieve this, the IFG employs selective attention to inhibit unnecessary information (Chong et al., 2008). In this study, because the participants were informed about distractors beforehand, they could easily suppress them from the beginning of problem-solving. The distractors were suppressed using the bilateral IFG, and this activity gradually decreased until the problem was resolved. In contrast, in the later stages of problem-solving, it becomes essential to suppress previously dominant but incorrect representations and establish a new perspective (Lv, 2015). This process may involve the DLPFC, which controls attention in a top-down manner (Anderson et al., 2009; Shen et al., 2013; Wu and Chen, 2021). Tang, using a kanji chunk decomposition task, reported that the right DLPFC becomes more activated with increased difficulty of chunk decomposition (Tang et al., 2016). However, participants initially needed to understand the problem to identify the information that should be suppressed; after that, they could inhibit the existing representation. In the present study, no such suppression occurred, and consequently, no differences in DLPFC activity were detected. These findings indicate that the IFG is primarily involved in low-level suppression tasks, such as filtering and selective attention, whereas the DLPFC is engaged in higher-level suppression, including forming new representations and inhibiting established but irrelevant ones. These findings suggest that the strategies and neural regions involved in suppressing unnecessary information differ between the initial and later stages of problem-solving.

This study had some limitations. We could not precisely determine when distractor suppression ended. While differences in brain activity were observed at problem onset, they disappeared by the solution stage, suggesting a convergence of cognitive processes. However, because problem-solving duration and processes vary across problems, it is challenging to define events that capture mid-phase neural activity. This highlights the limitations of event-related fMRI modeling in capturing continuous cognitive transition. To address this issue, approaches beyond event-based analyses must be explored.

In conclusion, this study demonstrated the role of the bilateral IFG in suppressing unnecessary information processing. The findings suggest that different suppression mechanisms are involved in the early and later stages of problem-solving. Suppression-related brain activity in the early stages appeared to gradually decrease until the problem was resolved. However, event-based models alone cannot precisely delineate the suppression of the endpoints.

## Data Availability

The raw data supporting the conclusions of this article will be made available by the authors, without undue reservation.

## Footnotes

1
http://marsbar.sourceforge.net
